# Supplementary material for: Epigenomics and transcriptomics of systemic sclerosis CD4+ T cells reveal long-range dysregulation of key inflammatory pathways mediated by disease-associated susceptibility loci
Source: Genome Med. 2020 Sep 25;12:81. doi: 10.1186/s13073-020-00779-6 (PMC7519528; doi:10.1186/s13073-020-00779-6)
Supplement: Supplementary file 1 — Additional file 1: Table S1. Clinical characteristics of cohort. [file 13073_2020_779_MOESM1_ESM.pdf]

Table S1. Clinical characteristics of cohort

	SSc (N = 48)		Controls (N = 16)		P-value
	Mean or %	SD or N	Mean or %	SD or N	
Age (years)	53.2	14.3	53.6	14.8	0.926
Female	91.7 %	44	75 %	12	0.081
Skin disease					
Sine Scleroderma (ssSSc)	18.7 %	9			
Limited (lcSSc)	41.7 %	20			
Diffuse (dcSSc)	31.3 %	15			
Early (eSSc)	8.3 %	4			
Smoking					
Current	12.5 %	6			
Past	27.1 %	13			
Never	39.6 %	19			
Lung disease					
None	47.9 %	23			
Interstitial lung disease	37.5 %	18			
Pulmonary hypertension	14.6 %	7			
Immunosuppressive medication	43.8 %	21			
Disease duration (years)	15.4	11.7			
Raynaud’s phenomenon	97.9 %	47			
Digital ulcers	50 %	24			
Telangiectasias	77.1 %	37			
Esophageal hypomotility	85.4 %	41			
Scleroderma renal crisis	4.2 %	2			
Tendinopathy	16.7 %	8			
Arthritis	14.6 %	7			
Myositis	18.8 %	9			
Calcinosis	29.2 %	14			
Anti-nuclear antibodies	100 %	48			
Anti-centromere	22.9 %	11			
Anti-topoisomerase I	20.8 %	10			
Anti-RNA polymerase III	12.5 %	6			
Anti-PM/Scl	10.4 %	5			
Immunosuppressive therapy[a]	43.8 %	21			
Vasodilator therapy[b]	29.2 %	14			

[a]Corticotherapy, azathioprine, mycophenolic acid, methotrexate, cyclophosphamide or rituximab.

[b]Antiendothelin receptor antagonist, prostanoids or phosphodiesterase-5 inhibitors
