# Supplementary material for: Epigenomics and transcriptomics of systemic sclerosis CD4+ T cells reveal long-range dysregulation of key inflammatory pathways mediated by disease-associated susceptibility loci
Source: Genome Med. 2020 Sep 25;12:81. doi: 10.1186/s13073-020-00779-6 (PMC7519528; doi:10.1186/s13073-020-00779-6)
Supplement: Supplementary file 3 — Additional file 3: Figure S1. Additional analyses of DNA methylation datasets. [file 13073_2020_779_MOESM3_ESM.pdf]

Figure S1

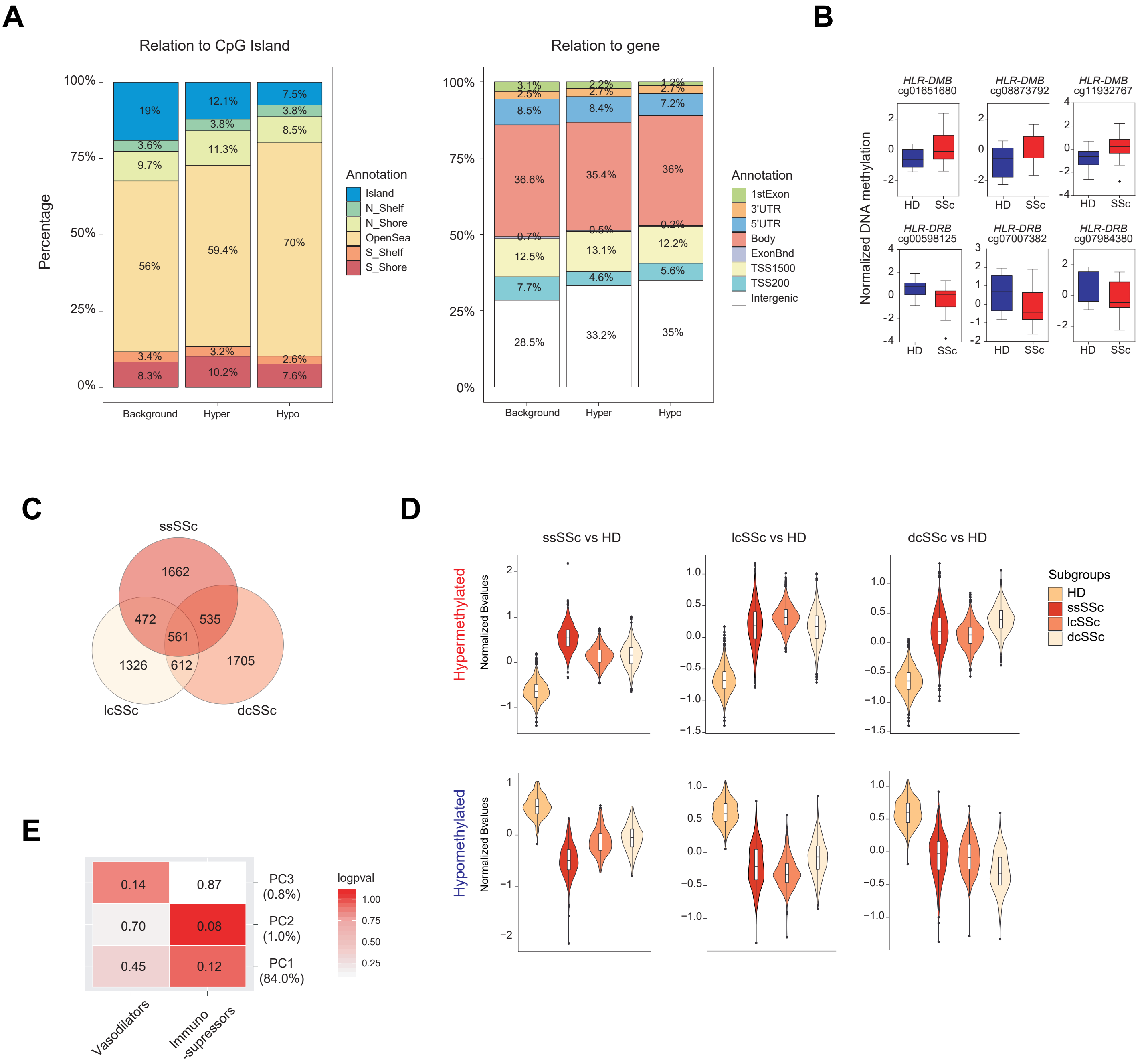

Additional file 3: Figure S1. (A) Distribution of DMPs in relation to CpG islands (left panel) and in relation to gene (right panel) according to annotations obtained from Infinium MethylationEPIC array. Proportion of each category was calculated for background, and hypo- and hypermethylated DMPs and represented as a percentage. Abbreviations: N - north; S - south; 1stExon - first exon; UTR - untranslated region; ExonBnd - exon boundary; TSS - transcription start site. (B) Representative graphs of normalized DNA methylation of relevant DMPs within the HLA gene cluster. (C) Overlap of differentially methylated CpGs identified by comparing limited (lcSSc), diffuse (dcSSc) and sine (ssSSc) with healthy controls. (D) Violin plots of normalized beta values of hyper- and hypomethylated CpGs identified in the comparisons lcSSc-HD, dcSSc-HD and ssSSc-HD. (E) Wilcoxon signed-rank test of variables in which patients were treated with vasodilators (n = 14) and/or immunosuppressive therapy (n = 21) and their association with the first three principal components (PC) identified for SSc-associated DMPs. Percentage represents the percentage of contribution of each PC to total variance.
