# Supplementary material for: Epigenomics and transcriptomics of systemic sclerosis CD4+ T cells reveal long-range dysregulation of key inflammatory pathways mediated by disease-associated susceptibility loci
Source: Genome Med. 2020 Sep 25;12:81. doi: 10.1186/s13073-020-00779-6 (PMC7519528; doi:10.1186/s13073-020-00779-6)
Supplement: Supplementary file 5 — Additional file 5: Figure S2. Additional analyses of DMRs and gene expression datasets. [file 13073_2020_779_MOESM5_ESM.pdf]

Figure S2

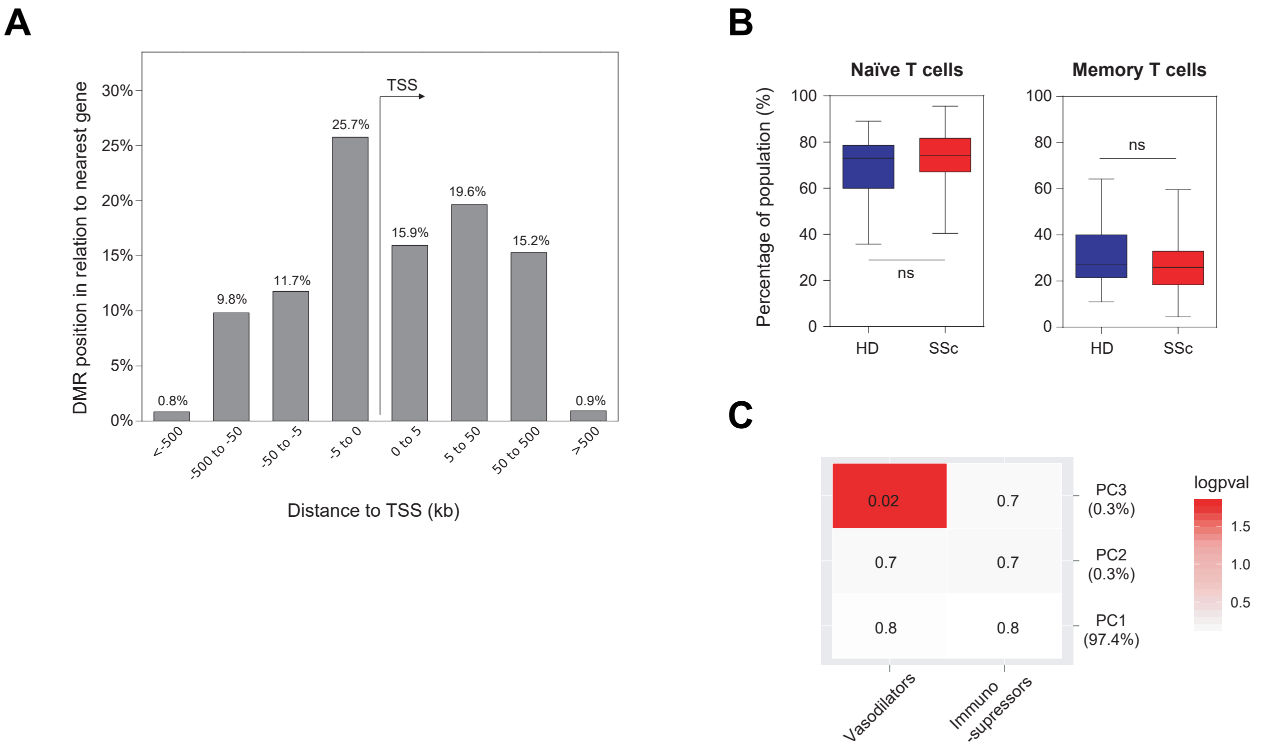

Additional file 5: Figure S2. (A) Bar graph representing the genomic distribution of identified DMRs in relation to transcription start site (TSS) as mapped utilizing the GREAT online tool. (B) Deconvolution analysis of CD4+ T cell gene expression utilizing the Absolute Immune Signal (ABIS) online tool. (C) Wilcoxon signed-rank test of variables in which patients were treated with vasodilators (n = 14) and/or immunosuppressive therapy (n = 21) and their association with the first three principal components (PC) identified for SSc-associated DEGs. Percentage represents the percentage of contribution of each PC to total variance.
