# Supplementary material for: Epigenomics and transcriptomics of systemic sclerosis CD4+ T cells reveal long-range dysregulation of key inflammatory pathways mediated by disease-associated susceptibility loci
Source: Genome Med. 2020 Sep 25;12:81. doi: 10.1186/s13073-020-00779-6 (PMC7519528; doi:10.1186/s13073-020-00779-6)
Supplement: Supplementary file 10 — Additional file 10: Figure S3. Association of DEG-DMP interactomes with SSc-associated susceptibility alleles. [file 13073_2020_779_MOESM10_ESM.pdf]

Figure S3

A

Chr	Locus	Independent variant	Effect allele	OR	*p*-value
5	*TNIP1*	rs3792783	G	1.20	2.42E-12
15	*CSK*	rs1378942	C	1.18	1.84E-14
17	*GSDMB*	rs9303277	C	0.89	9.88E-08
19	*IL12RB1*	rs2305743	A	0.83	4.64E-10

B

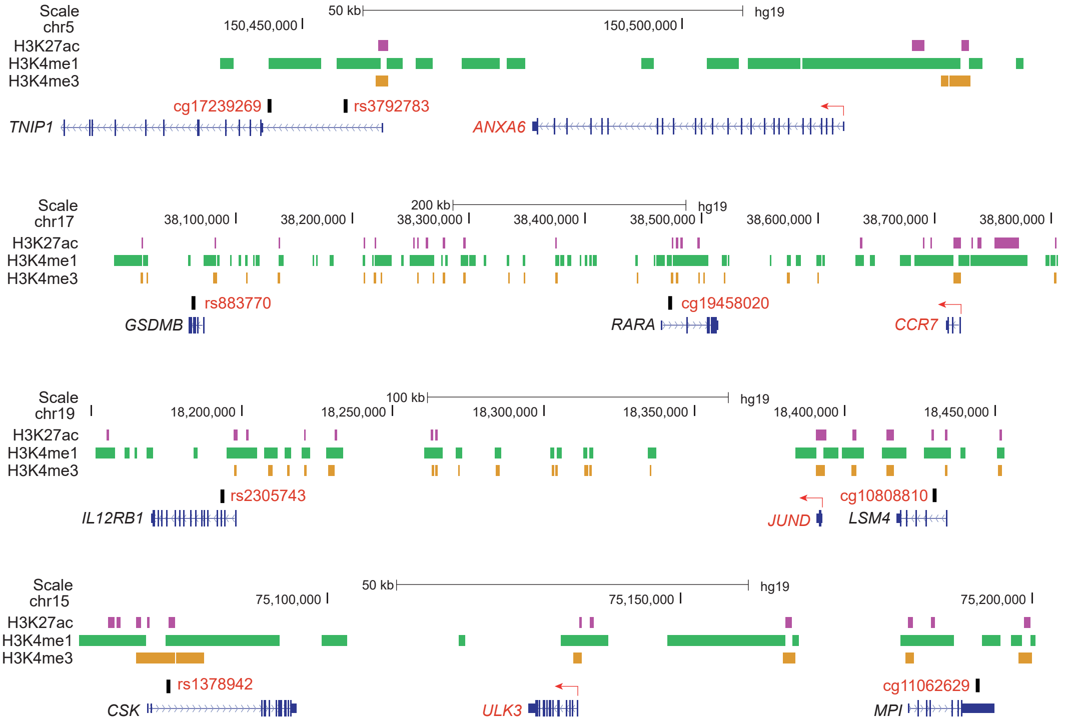

C

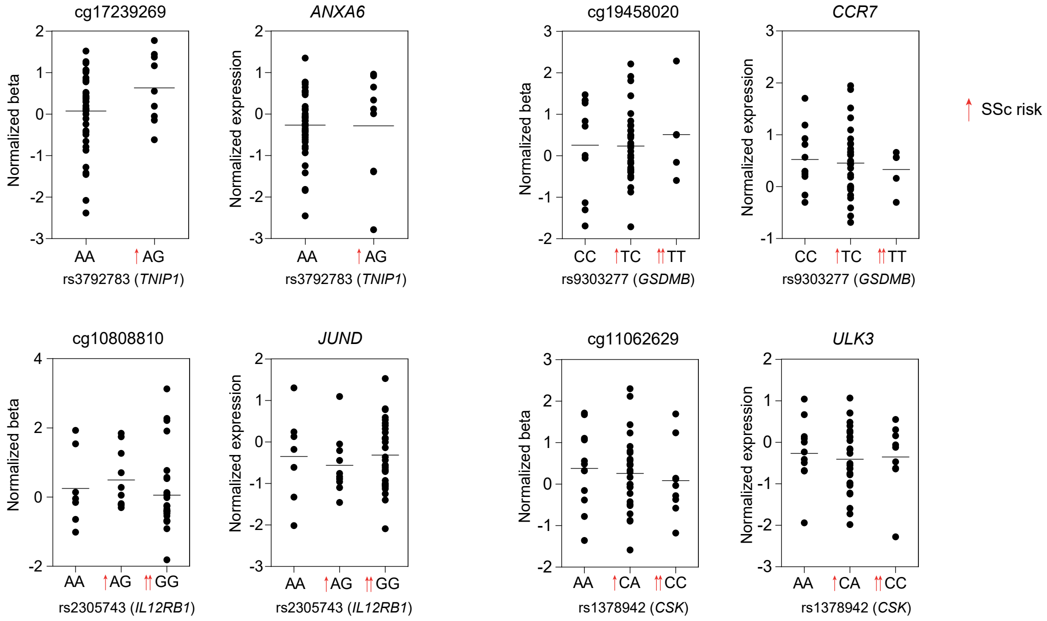

Additional file 10: Figure S3. (A) Table summarizing odds ratios and p-values of SSc risk variants identified by López-Isac et al. [15]. (B) ChIP-seq datasets obtained from total CD4+ T cells of H3K27ac, H3K4me1 and H3K4me3 downloaded from the BLUEPRINT database. UCSC genome browser representations of significant signals within the vicinity of interactomes rs3792783-cg17239269-ANXA6, rs883770-cg19458020-CCR7, rs2305743-cg10808810-JUND and rs1378942-cg1162629-ULK3. (C) DNA methylation of DMPs and gene expression of interacting DEGs of SSc patients separated by the presence of previously identified risk alleles annotated in (A).
